# Transforming Healthcare: Mozambique’s Pioneering Integrative Medicine Course

**DOI:** 10.5334/aogh.4785

**Published:** 2026-02-12

**Authors:** Delfina Hlashwayo, Filomena Barbosa, Angelina Martins, Tufária Mussá, Amélia Furvela, Telma Magaia, Felda Langa, Natércia Madeira, Esperança Rafael, Eliette Munezero, Nurah Virahsawmy, Marta Maculuve, Alice Massingue

**Affiliations:** 1 Faculty of Sciences, Eduardo Mondlane University, Maputo, Mozambique; 2 Ministry of Health, Mozambique; 3 Faculty of Medicine, Eduardo Mondlane University, Maputo, Mozambique

**Keywords:** integrative medicine, medical education, health students, Mozambique

## Abstract

*Introduction:* This manuscript evaluates the outcomes of a pioneering Integrative Medicine Course implemented at Eduardo Mondlane University, aiming to strengthen medical and health students’ knowledge and skills while fostering a holistic approach to patient care. Delivered in two editions—an intensive in‑person program and an extended online format—the course sought to improve understanding of integrative medicine.

*Methods:* A mixed‑methods approach was adopted, combining data from participatory observation, pre‑ and post‑course surveys, focus group discussions, and final course evaluations. Self‑assessments of knowledge and skills were collected before and after the course. Final exam results were analyzed to assess knowledge acquisition. The in‑person edition was conducted over 1 week (September 16–20, 2024), and the online edition spanned 8 weeks (January–February 2025). Both formats included weekly lectures, practical sessions, and interactive discussions. The Wilcoxon signed‑rank test was used to evaluate changes in knowledge and skills.

*Results and discussion:* A total of 164 students enrolled, with 134 completing the course (completion rate of 82%). Most participants were female (81%), with an average age of 23 years (SD ± 3.7); 61% were medical students, and three were postgraduate students. Satisfaction was high, with 66% awarding the highest rating. The most highly rated aspects included instructors (78%), course organization (77%), and resources provided (75%). Phytotherapy emerged as the most relevant topic, followed by traditional medicine, mental well‑being, and nutrition. The participatory teaching approach was preferred, accounting for 63% of mentions. Post‑course evaluations showed significant improvements in knowledge, interest, attitudes, and competencies (*p* < 0.05). All students passed the final exam, with an average score of 18/20.

*Conclusions:* The course successfully enhanced students’ understanding and application of integrative medicine. Both delivery formats proved effective in engaging learners and fostering critical skills. This initiative establishes a foundation for advancing integrative medicine education and research in Mozambique.

## Introduction

Mozambique, a country in southeastern sub‑Saharan Africa, is home to approximately 35 million people and is characterized by a diverse cultural heritage and rich biodiversity. Its healthcare system faces significant challenges, balancing a high burden of infectious diseases with a rising prevalence of noncommunicable conditions [1]. HIV/AIDS remains the leading cause of mortality, while tuberculosis, malaria, and respiratory infections continue to be major public health concerns [2].

Despite ongoing efforts to strengthen the healthcare system, access to medical services remains limited, with only 47% of the population having direct access to healthcare facilities. As a result, many Mozambicans rely on traditional medicine to meet their health needs [3–9], reflecting both necessity and deeply rooted cultural practices [9]. However, alongside this widespread reliance—particularly in rural areas—a different scenario is emerging in urban centers. In cities such as Maputo, access to hospitals and private clinics is more common, although public facilities are often overcrowded and face resource constraints. Notably, some public hospitals have begun integrating practices such as acupuncture into their services, and several Chinese medicine clinics operate in the capital, attracting a growing clientele.

In addition, interest in complementary and alternative medicine is expanding, particularly among urban populations with increased access to information and global health trends. Aromatherapy, for instance, has gained popularity, with growing sales and online engagement. Modalities that were previously unfamiliar—such as meditation and mind–body practices—are increasingly visible and sought after. In the absence of structured integrative care services, many individuals navigate a fragmented therapeutic landscape, combining conventional and alternative strategies on their own.

Integrative medicine, which combines conventional medical approaches with evidence‑based complementary and traditional therapies, is increasingly recognized worldwide for its potential to enhance patient‑centered care [10]. This holistic approach emphasizes the interconnectedness of physical, mental, and social well‑being, incorporating diverse healing modalities such as herbal medicine, acupuncture, meditation, and mind–body practices. Despite this evolving landscape, in Mozambique, integrative medicine remains largely absent from formal medical education, national health policy, and clinical practice. There has been little public discourse or institutional positioning around its potential. Yet, the growing interest and scattered availability of such practices highlight both a demand and an opportunity. Advancing integrative medicine in Mozambique holds potential to promote more person‑centered care, enhance health outcomes, and foster greater alignment between biomedical services and the diverse therapeutic practices already embedded in local contexts, while also encouraging interdisciplinary collaboration.

Establishing structured training programs is essential for building capacity, increasing awareness, and fostering a deeper understanding of integrative medicine among students and healthcare professionals. This initiative not only strengthens academic and clinical competencies but also encourages dialogue on integrative healthcare in Mozambique, paving the way for its broader recognition and potential inclusion in both undergraduate and postgraduate education.

At Eduardo Mondlane University (UEM)—Mozambique’s oldest and largest public university, and the leading institution for the training of physicians and other health professionals, including those in biomedical sciences—there is currently no specific course or discipline dedicated to traditional, complementary, or alternative medicine within the formal curricula of medical students. This gap limits the ability of future healthcare professionals to effectively engage with the complementary and alternative practices that are already deeply embedded in the healthcare choices of the population. To bridge this gap, UEM has launched the first tuition‑free Integrative Medicine Training Course, leveraging its expertise in biomedicine, complementary and alternative medicine, ethnopharmacology, and related disciplines.

The Faculty of Sciences, which offers undergraduate and postgraduate programs—including a doctoral program in Biosciences and Public Health—was responsible for administering the course. As a leading center for biomedical education and research, the faculty brings together experts in ethnobotany, ethnopharmacology, nutrition, and traditional medicine, among other fields. This multidisciplinary expertise provided a strong foundation for the Integrative Medicine Training Course, ensuring a comprehensive and evidence‑based approach to the subject. This initiative was made possible through a successfully secured grant of $28,500 USD awarded by the Weil Foundation (USA).

The primary objective of this pioneering initiative was to equip medical students, as well as students from other health‑related fields, with a comprehensive understanding of integrative medicine. The program extended this opportunity to students across multiple institutions with the goal of cultivating a new generation of healthcare professionals proficient in both conventional and integrative approaches, while also fostering interest in research within this field. This manuscript details the implementation, impact, and key outcomes of the first Integrative Medicine Training Course at Eduardo Mondlane University in Mozambique.

## Methods

This study employed a mixed‑methods approach, integrating data from participatory observation, pre‑ and post‑course surveys, focus group discussions, and final course evaluations. The surveys collected sociodemographic information, participants’ motivations for enrolling, and self‑assessments of their competencies before and after the training. Additionally, final exam results were analyzed to assess knowledge acquisition. The protocol for evaluating the course’s impact was approved by the Faculty of Medicine of Eduardo Mondlane University and the Maputo Central Hospital Joint Institutional Committee on Bioethics for Health (Approval No. CIBSFM&HCM/66/2024).

### Course organization

#### 1. Initial planning

**Figure 1:**
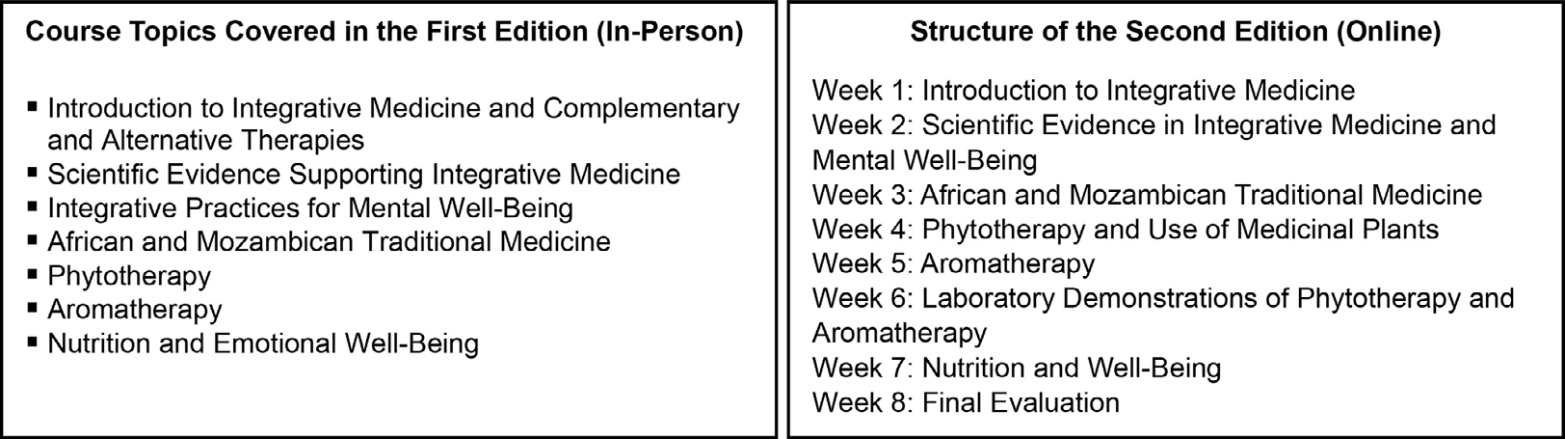
Overview of the course structure: in‑person and online editions.

The planning phase from June to July 2024 involved coordination meetings between the course organizers and the Faculty of Medicine leadership at UEM to discuss implementation strategies. Given the academic schedule of medical students, it was determined that the course would be offered as a 1‑week intensive program, held in multiple cohorts. The first edition was scheduled for September 16–20, during the mid‑semester break when no regular classes were in session at UEM. The training ran daily from 08:00 to 13:00, totaling 25 h. Following this initial planning phase, key topics were identified, as outlined in Figure 1.

In consultation with faculty members, the course program was finalized and submitted for approval, and specialized instructors with significant expertise in each topic were invited to lead the respective modules.

#### 2. Preparations, course promotion, and implementation

To facilitate hands‑on learning experiences, various laboratory supplies and equipment were procured from July to September 2024, including equipment for essential oil extraction, chemical compound extraction, and phytochemical analysis of medicinal plants. These resources enabled students to engage in practical applications of integrative medicine that were not previously available in the faculty’s laboratory. As part of the outreach strategy, the course was widely advertised at the Faculty of Sciences, Faculty of Medicine, and other universities, including *Instituto Superior de Ciências de Saúde* (ISCISA) and *Instituto Superior de Ciências e Tecnologia de Moçambique* (ISCTEM), as well as through LinkedIn, WhatsApp, and Instagram.

Following the successful implementation of the first edition, the course coordination team assessed its effectiveness and determined that the same format could be replicated for future cohorts. The next three editions were initially scheduled for December 2024 and January 2025 to accommodate students during the holiday period. However, from October 2024 to early 2025, Mozambique experienced increasing political and social tensions, leading to mobility restrictions and insecurity. In response, and with the approval of the funding agency, the course was transitioned to an online format. The final three editions were consolidated into a single online cohort, including all eligible candidates who met the inclusion criteria.

The second edition of the course, delivered online, was structured as an 8‑week program to provide students with more time to consolidate the material and to accommodate their full‑ or part‑time academic commitments. The overall structure is outlined in Figure 1, and the detailed program for both the first and second editions of the course is provided in Supplementary File 1.

Weekly content included recorded lectures with an average time of 1 h, as well as discussion forums, practical activities, and quizzes. Additionally, a live 40‑min session was held every Friday at 19:00 to review and discuss key concepts. The course was hosted on Google Sites, with Slido (https://www.slido.com/) integrated for quizzes and discussion forums. A WhatsApp group was created to facilitate communication, and weekly instructional emails were sent to participants. Course materials were made available through a shared Google Drive, which was embedded within the course website, and students were required to complete a short weekly assignment, such as discussing a specific topic and proposing practical solutions.

Participants in both editions of the course were selected based on predefined eligibility and prioritization criteria. Preference was given to undergraduate students in medicine or health‑related fields who demonstrated strong motivation in their application forms and confirmed availability. Applicants were excluded if they were not enrolled in academic programs, were outside the health or life sciences fields, submitted incomplete applications, or failed to respond during follow‑up.

#### 3. Assessment and certification

In both the in‑person and online editions, a final evaluation was conducted, consisting of 20 multiple‑choice questions worth 20 points. A minimum score of 10 was required to pass. Upon successful completion, students received a certificate signed and stamped by the Director of the Faculty of Sciences.

### Data analysis

Data collection procedures varied across editions. In the first edition, most responses were obtained via online forms, but printed versions were provided to participants who had not completed the online surveys. Additionally, the post‑course questionnaire and final evaluation in this edition were administered exclusively in printed format. In the second edition, all data were collected through online forms. All responses were compiled and managed using Microsoft Excel 2021. Data validation checks were conducted to ensure internal consistency and completeness before analysis.

Descriptive analyses were performed to characterize the participants’ sociodemographic profile, including absolute frequencies, proportions, and total counts. To evaluate changes in self‑reported knowledge and skills, responses to identical pre‑ and post‑course questions—measured using a 5‑point Likert scale (1 = very poor to 5 = very good)—were compared. The distribution of the data was assessed to determine the appropriate statistical tests. As the data did not follow a normal distribution, non‑parametric methods were applied. The Wilcoxon signed‑rank test was used to compare pre‑ and post‑course scores, with a significance threshold of *p* < 0.05. All statistical analyses were performed using R software, version 4.4.3 for Windows.

Comparative analyses were conducted separately for the first (in‑person) and second (online) editions, as well as for the combined dataset. Final evaluation scores were summarized using measures of central tendency (mean, median) and dispersion (minimum, maximum). Satisfaction‑related items were represented using 100% stacked bar charts to facilitate interpretation.

## Results

Both editions of the Integrative Medicine Course were successfully implemented and generated significant interest among students from diverse academic backgrounds. For the first edition, a total of 239 applications were received (including a small number of duplicates). Given the limited number of available seats, 30 students were selected. For the subsequent cohort, which consolidated three planned editions into a single online version, 185 new applications were received, in addition to 54 applicants from the first edition who had not been selected. A total of 134 students were enrolled in the second cohort. Of the 30 students in the first edition, 29 completed the course, while in the second edition, 105 students completed the course. In total, 134 students completed the Integrative Medicine Course.

### Participant characteristics

A total of 81% of the participants were female, with an average age of 23 years (SD ± 3.7). A total of 98% self‑identified as Black, and 94% were single. Students represented ten higher education institutions, with Eduardo Mondlane University contributing 48% of all participants. In terms of academic programs, 61% were enrolled in a degree in medicine, followed by a bachelor’s degree in biology and health (16%). While 131 participants were undergraduate students, two were enrolled in master’s programs and one in a doctoral program. A total of 53% of the students were in their third or fourth year of study. Table 1 presents the demographic and academic characteristics of the 134 participants across both editions.

**Table 1:** Demographic and academic characteristics of participants in the Integrative Medicine Course (first and second editions).

CATEGORY	FIRST EDITION (*N* = 29)	SECOND EDITION (*N* = 105)	TOTAL (*N* = 134)
**Gender**			
Male	7 (24%)	19 (18%)	26 (19%)
Female	22 (76%)	86 (82%)	108 (81%)
**Age** (average ± SD)	23 ± 3.9	23 ± 3.6	23 ± 3.7
**Race**			
Black	29 (100%)	102 (97%)	131 (98%)
Indian	0 (0%)	3 (3%)	3 (2%)
**Civil status**			
Single	27 (93%)	99 (94%)	126 (94%)
Stable union	1 (3%)	1 (1%)	2 (1%)
Married	1 (3%)	5 (5%)	6 (5%)
**Religion**			
Evangelical Christian	9 (31%)	43 (41%)	52 (39%)
Catholic Christian	11 (38%)	34 (32%)	45 (34%)
Islamic	4 (14%)	13 (12%)	17 (13%)
Other Christians	5 (17%)	11 (11%)	16 (12%)
No religion	0 (0%)	4 (4%)	4 (3%)
**Main higher education institutions**			
UEM	21 (72%)	43 (41%)	64 (48%)
ISCTEM	5 (17%)	40 (38%)	45 (34%)
ISCISA	2 (7%)	10 (10%)	12 (9%)
USTM	0 (0%)	5 (5%)	5 (4%)
Others	1 (4%)	7 (7%)	8 (6%)
**Enrolled academic program**			
Medicine degree	24 (83%)	58 (55%)	82 (61%)
BSc in Biology and Health	2 (7%)	20 (19%)	22 (16%)
BSc in Public Health	0 (0%)	5 (5%)	5 (4%)
BSc in Psychology	1 (3%)	4 (4%)	5 (4%)
BSc in Biomedical Laboratory Technology	0 (0%)	5 (5%)	5 (4%)
BSc in Nutrition	1 (3%)	3 (3%)	4 (3%)
Others	1 (3%)	10 (10%)	11 (8%)
**Year of study**			
1–2	17 (59%)	18 (17%)	35 (26%)
3–4	8 (28%)	63 (60%)	71 (53%)
5–6	4 (14%)	24 (23%)	28 (21%)
**Previous experience in healthcare**			
No	15 (52%)	56 (53%)	71 (53%)
Yes	14 (48%)	49 (47%)	63 (47%)
**Previous training in CAM**			
No	26 (90%)	68 (65%)	94 (70%)
Yes	3 (10%)	37 (35%)	40 (30%)
**Previous use of CAM**			
No	17 (59%)	73 (70%)	90 (67%)
Yes	12 (41%)	32 (30%)	44 (33%)

In total, 47% of participants reported some prior experience in health‑related activities, most commonly through internships (mentioned by 42 participants). Regarding prior training in complementary or alternative medicine (CAM), 30% reported having received some form of instruction, with the most frequent being dietary supplements (14%) and meditation (8%). In total, 33% reported prior use of CAM therapies, with the most common being dietary supplements (12%), meditation (10%), and phytotherapy (9%). Among those who had used any CAM approach (*n* = 44), 35 stated that it had been beneficial. Expanded data on academic institutions, programs, and CAM training modalities are provided in Supplementary File 2.

None of the students had previously participated in a course specifically dedicated to integrative medicine. The most frequently mentioned areas of future specialization included general surgery (and its subfields; total of 24 mentions), gynecology (18 mentions), and public health (11 mentions). Open‑ended responses regarding motivations to enroll in the course indicated strong interest and curiosity about the topic, as well as a desire to acquire new knowledge and skills related to integrative approaches in healthcare. When asked what they hoped to learn during the course, participants provided diverse responses, with a common emphasis on acquiring a deeper understanding of integrative medicine and the various complementary and alternative therapies it encompasses.

### Satisfaction with the course

Throughout both editions, there was significant interaction and satisfaction among participants. In the first edition, the sessions were highly dynamic, with students actively sharing their experiences and perspectives, which greatly enriched the learning process. Both theoretical and practical lessons were highly interactive. Attendance was consistent, and students demonstrated great enthusiasm.

The online approach allowed us to reach students beyond Maputo and accommodate a larger number of participants. The incorporation of engagement mechanisms, such as weekly tasks, quizzes, interactive platforms, and direct communication with instructors, fostered active participation. Both in‑person and online formats proved valuable, each with areas for improvement. A key limitation of the online format was the inability to conduct practical sessions in the laboratory; to address this, we recorded these lab sessions and made them available on the course website to participants. Additionally, some theoretical activities, such as case studies and hands‑on exercises, which were effectively implemented in person, were more challenging in the online setting. Nonetheless, the results demonstrated that online delivery is a viable and effective alternative. Despite the limited time to replan the course for an online format, we leveraged the available tools to ensure engagement, maintain students’ interest, and provide a user‑friendly learning platform.

**Figure 2:**
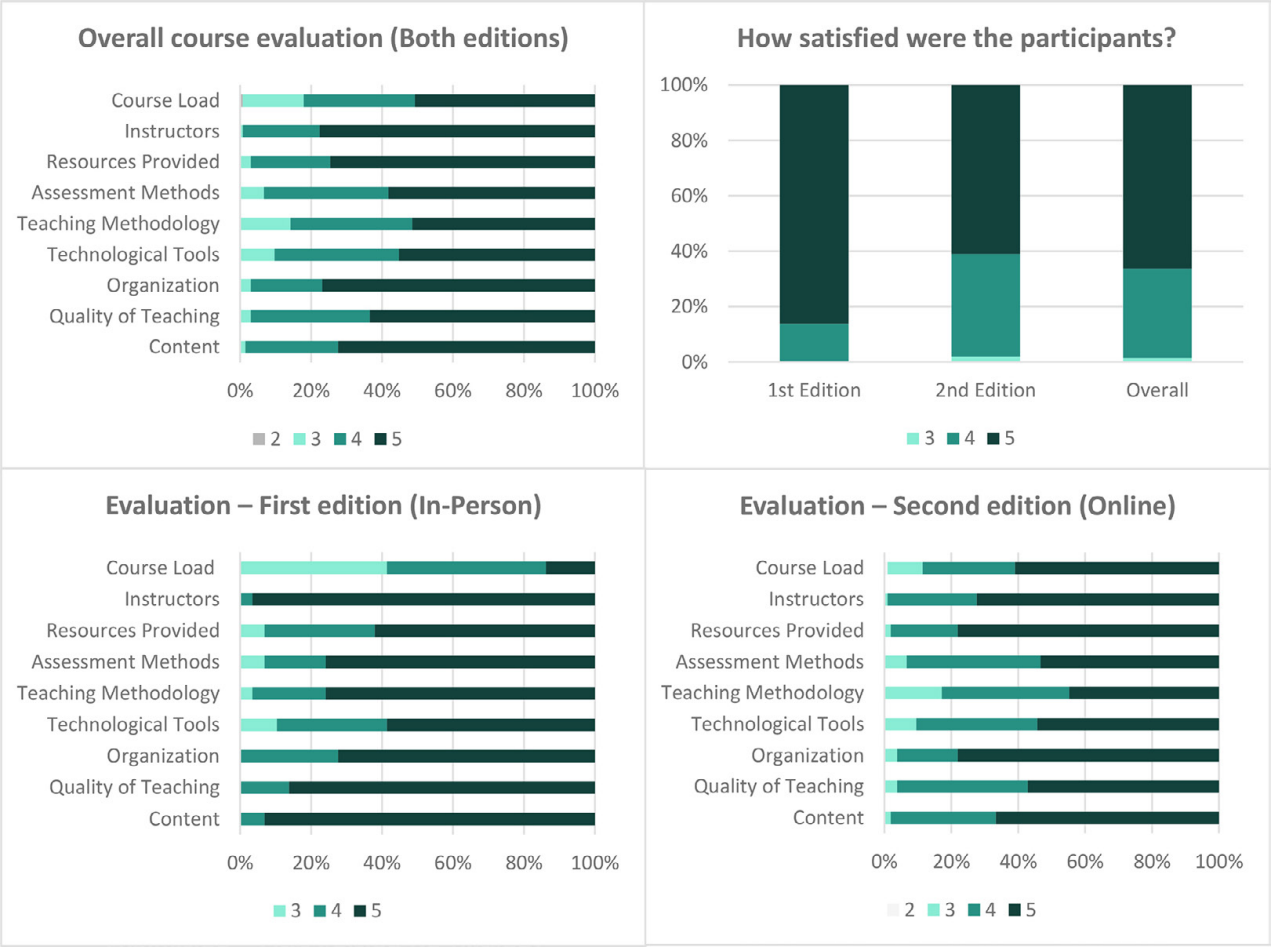
Course satisfaction ratings: an overview of overall scores and a detailed comparison by the first and second editions.

Participants’ satisfaction reflected a predominantly positive perception, with 66% (89) of students giving the highest rating of 5, while 32% (43) rated it 4. Only two students (2%) assigned a rating of 3 (Figure 2). One of the main concerns raised by the students of the first edition was the course workload. They expressed great enthusiasm for the subjects and wanted more time to explore them. As a result, in the first edition, 41% rated the workload as 3, whereas in the second edition, this proportion decreased to 11%, rating it below 4, indicating that the modular structure over 8 weeks led to an improvement.

Overall, the highest rated satisfaction parameters on the Likert scale (5) were the instructors (78%), organization (77%), resources provided (75%), and content (72%). In the first edition, the instructors received the highest rating from 96.6% of students, followed by course content at 93% and teaching quality at 86%. In the second edition, the organization and provided resources each received the highest rating from 61% of students in both categories.

Regarding course content, phytotherapy was the topic that participants considered most relevant, with 43 mentions. Other frequently highlighted topics included traditional medicine (28 mentions), as well as mental well‑being and nutrition (both with 27 mentions). Supplementary File 2 presents a complete summary.

In response to the question regarding potential additions or removals of topics, 19% (26 students) suggested changes. This corresponds to 41% (12) in the first (in‑person) edition and 13% (14) in the second (online) edition. The main recommendations from the first edition emphasized expanding the coverage of mind–body practices and complementary therapies, particularly phytotherapy, mindfulness, music therapy, physical exercise, and spirituality. For the online edition, participants also suggested increasing interactivity, shortening the introductory module, and incorporating more practical sessions. Further suggestions included exploring the role of integrative medicine in the treatment of substance use disorders, pandemic response, and disease management in African contexts. Additionally, students proposed encouraging dialogue between traditional and conventional practitioners and the development of dedicated courses for each major topic covered.

In terms of teaching methods across both editions, there was a clear preference for more active and participatory learning (63% of mentions). Lectures were also appreciated, accounting for 34% of mentions in both groups, including the online edition.

**Figure 3:**
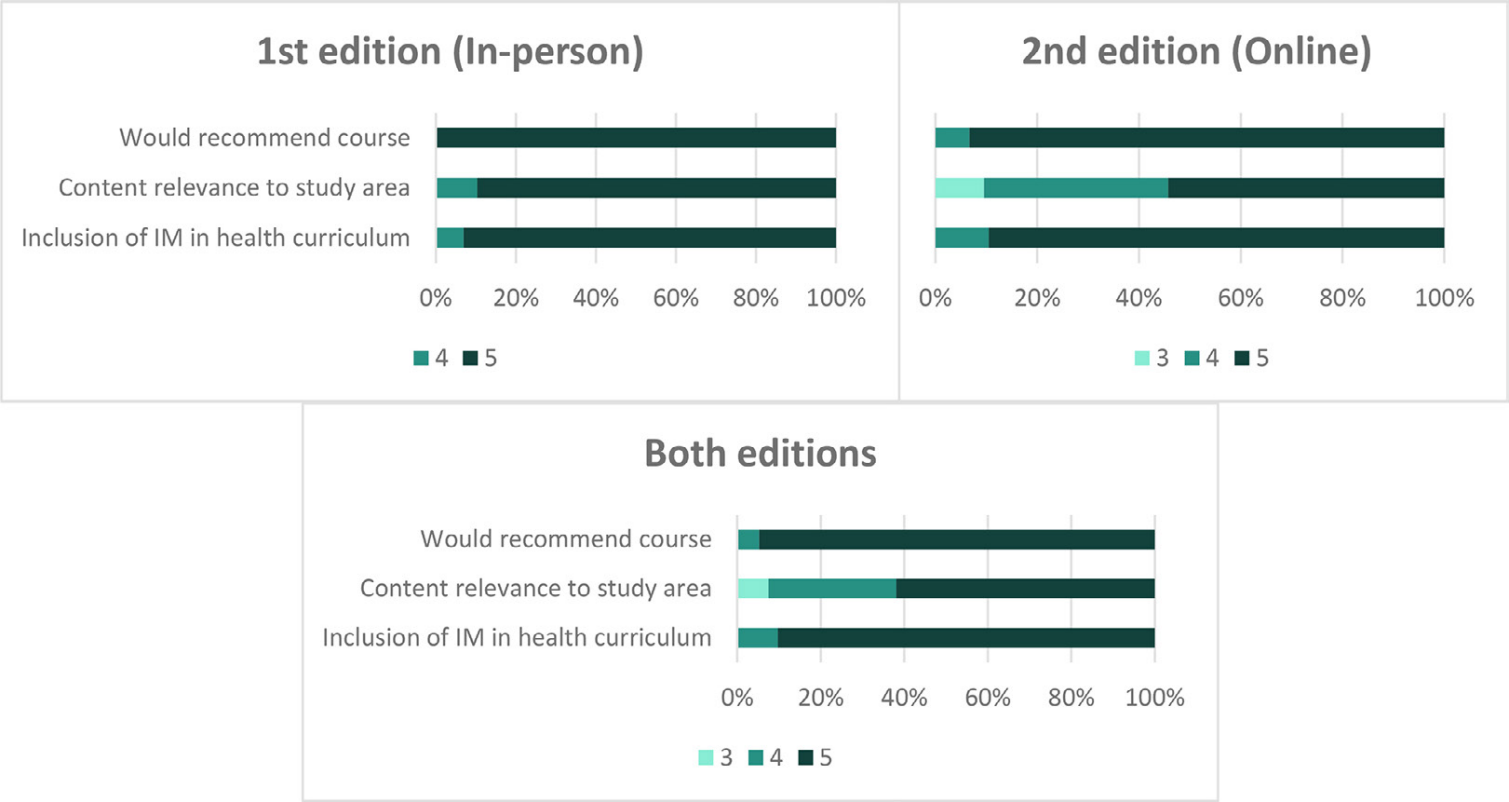
Student ratings on relevance, importance, and recommendation of the course.

When asked about recommending the course and the relevance of integrating integrative medicine into health education, all responses were rated 3 or higher on the Likert scale. Specifically, 95% of participants would highly recommend the course (rated 5). In terms of content relevance, 62% of participants considered the course highly relevant to their field of study or practice. Furthermore, 90% of participants rated the inclusion of integrative medicine in the health curriculum as highly important, also giving it a score of 5 (Figure 3).

In response to the open‑ended question about the advantages the Integrative Medicine Course will bring to participants’ careers, many highlighted the ability to approach patient care holistically and to demystify aspects of complementary and alternative medicine. Participants emphasized the value of adopting a comprehensive approach to healthcare, combining both traditional and modern practices to better address patient needs.

Regarding the final question about any other comments or suggestions, many responses expressed gratitude for the course’s organization, the depth of learning, and the overall educational experience. Students appreciated the structured nature of the course and the knowledge it provided into various integrative health practices.

### Comparison of pre‑ and post‑course self‑perceived integrative medicine‑related attitudes, perceptions, interest, and skills

A significant increase (*p* < 0.05) in all parameters between pre‑ and post‑course assessments was observed, except for the perceived strength of the scientific evidence supporting the clinical benefits of integrative medicine (Table 2). For this parameter, there was a significant decrease post‑course, which likely reflects students’ realization that, in many cases, traditional Mozambican medicine still lacks substantial scientific validation, even though other practices do have a stronger evidence base. It appears that students may have focused more on this aspect during the course.

**Table 2:** Pre‑ and post‑course self‑perceived integrative medicine‑related attitudes, perceptions, interest, and skills (scores range from 1 to 5).

	FIRST EDITION (*N* = 29)	SECOND EDITION (*N* = 105)	TOTAL (*N* = 134)	*P* VALUE
**Attitudes, perceptions, and interest in integrative medicine**				
1. Will this course be useful in your future professional practice in healthcare?	Pre: 4.83 ± 0.38 Post: 4.93 ± 0.26	Pre: 4.30 ± 0.66 Post: 4.60 ± 0.55	Pre: 4.41 ± 0.65 Post: 4.67 ± 0.52	*P*1 = 0.2986 *P*2 = 0.0002228 *P*3 = 0.0001138
2. How well do you think you know integrative medicine?	Pre: 1.97 ± 1.32 Post: 3.59 ± 0.57	Pre: 1.87 ± 0.81 Post: 3.61 ± 0.61	Pre: 1.89 ± 0.94 Post: 3.60 ± 0.60	*P*1 < 0.0001 *P*2 < 0.0001 *P*3 < 0.0001
3. What is your interest in integrative medicine?	Pre: 4.62 ± 0.68 Post: 4.83 ± 0.38	Pre: 4.45 ± 0.66 Post: 4.72 ± 0.53	Pre: 4.49 ± 0.67 Post: 4.75 ± 0.50	*P*1 = 0.1446 *P*2 = 0.00161 *P*3 = 0.00045
***Attitudes towards integrative medicine***				
4. Do you believe integrative medicine can significantly improve patient medical conditions?	Pre: 4.66 ± 0.55 Post: 4.86 ± 0.35	Pre: 4.46 ± 0.56 Post: 4.65 ± 0.54	Pre: 4.5 ± 0.56 Post: 4.69 ± 0.51	*P*1 = 0.09534 *P*2 = 0.008336 *P*3 = 0.001809
5. Do you believe integrative medicine could harm patients?	Pre: 1.62 ± 0.98 Post: 2.10 ± 1.21	Pre: 1.44 ± 0.78 Post: 1.80 ± 0.81	Pre: 1.48 ± 0.83 Post: 1.87 ± 0.92	*P*1 = 0.03917 *P*2 = 0.0005403 *P*3 < 0.0001
6. Do you believe there is a strong scientific evidence base for the clinical benefits of integrative medicine?	Pre: 3.93 ± 1.03 Post: 3.24 ± 1.18	Pre: 3.72 ± 0.89 Post: 3.56 ± 1.02	Pre: 3.77 ± 0.93 Post: 3.49 ± 1.06	*P*1 = 0.006936 *P*2 = 0.1739 *P*3 = 0.009705
7. Would you recommend integrative medicine practices to your future patients?	Pre: 4.34 ± 0.86 Post: 4.59 ± 0.63	Pre: 4.21 ± 0.66 Post: 4.46 ± 0.67	Pre: 4.24 ± 0.71 Post: 4.49 ± 0.66	*P*1 = 0.1778 *P*2 = 0.00249 *P*3 = 0.001002
8. Should integrative medicine be an option available in hospitals and clinics?	Pre: 4.52 ± 0.74 Post: 4.76 ± 0.51	Pre: 4.36 ± 0.70 Post: 4.52 ± 0.65	Pre: 4.40 ± 0.70 Post: 4.57 ± 0.63	*P*1 = 0.0593 *P*2 = 0.04747 *P*3 = 0.009757
***Integrative medicine skills***				
9. Ability to discuss the use of integrative medicine with others	Pre: 2.69 ± 1.34 Post: 4.07 ± 0.53	Pre: 2.57 ± 1.02 Post: 3.85 ± 0.68	Pre: 2.60 ± 1.09 Post: 3.90 ± 0.65	*P*1 = 0.0001853 *P*2 < 0.0001 *P*3 < 0.0001
10. Ability to communicate effectively with individuals who express an “alternative” health belief model	Pre: 3.45 ± 1.21 Post: 4.21 ± 0.56	Pre: 3.08 ± 0.87 Post: 3.87 ± 0.65	Pre: 3.16 ± 0.96 Post: 3.94 ± 0.65	*P*1 = 0.009804 *P*2 < 0.0001 *P*3 < 0.0001
11. Ability to refer individuals for an informed integrative medicine consultation	Pre: 3.34 ± 1.52 Post: 4.38 ± 0.68	Pre: 3.00 ± 1.07 Post: 3.99 ± 0.73	Pre: 3.08 ± 1.19 Post: 4.07 ± 0.73	*P*1 = 0.003014 *P*2 < 0.0001 *P*3 < 0.0001
12. Evaluation of the effectiveness of integrative medicine treatments	Pre: 3.41 ± 1.05 Post: 3.79 ± 0.82	Pre: 2.97 ± 0.99 Post: 3.59 ± 0.76	Pre: 3.07 ± 1.01 Post: 3.63 ± 0.77	*P*1 = 0.09031 *P*2 < 0.0001 *P*3 < 0.0001
13. Evaluation of the safety and/or risks of integrative medicine treatments	Pre: 3.10 ± 1.21 Post: 3.69 ± 0.97	Pre: 2.77 ± 1.07 Post:3.42 ± 0.79	Pre: 2.84 ± 1.10 Post: 3.48 ± 0.77	*P*1 = 0.05813 *P*2 < 0.0001 *P*3 < 0.0001

*P*1 = *p*‑value for pre‑ and post‑comparison for the first edition.

*P*2 = *p*‑value for pre‑ and post‑comparison for the second edition.

*P*3 = *p*‑value for pre‑ and post‑comparison for both editions combined.

Nonetheless, the overall results confirm that the course effectively enhanced the students’ understanding and engagement with integrative medicine.

All participants successfully passed the final assessment, with overall average scores being excellent, reaching an average of 18 (Table 3). This outcome further underscores the effectiveness of the course in enhancing participants’ comprehension of integrative medicine.

**Table 3:** Final assessment scores for the Integrative Medicine Course by edition.

EDITION	AVERAGE ± SD	MIN	MAX	MEDIAN
First edition (*n* = 29)	17.1 ± 1.78	13	20	17
Second edition (*n* = 105)	18.45 ± 1.78	12	20	19
Total (*n* = 134)	18.16 ± 1.86	12	20	19

## Discussion

This Integrative Medicine Course represents a significant milestone, being the first of its kind in Mozambique. It attracted a substantial number of applicants, including students from various disciplines—some not directly linked to health sciences—and even from outside the country, such as from the University of Algarve. The high number of applications, combined with the strong interest expressed in the application forms, reflects a growing demand for training in this area, not only among students but also among practicing health professionals.

From a total of 164 enrolled participants across all editions, 134 completed the course, corresponding to a completion rate of 81.7%, which is considered satisfactory for short‑term training programs. The demographic data align with the expected profile of health sciences students. A significant portion of participants were medical students, a strategic target group, as they are likely to apply these learnings in their clinical practice and help reshape the health system’s approach to integrative care. The course also included postgraduate students and, through the online format, extended its reach to participants outside of Maputo and even beyond the borders of Mozambique.

Most participants reported a high level of satisfaction with the course, both in‑person and online, and demonstrated a clear understanding of the relevance of integrative medicine in both their professional paths and personal lives. The assessments and final exam indicated measurable improvements in their knowledge and skills, confirming the educational effectiveness of the course.

The in‑person component adopted dynamic and participatory teaching methods. The sessions were carefully designed to avoid passive learning. For instance, in the phytotherapy module, students compared a monograph from the World Health Organization (WHO) with a Mozambican monograph, identifying gaps and opportunities for strengthening national monographs. In the traditional medicine module, role‑play exercises simulated clinical encounters in which patients disclosed the use of traditional therapies, encouraging students to reflect on how health professionals should respond. The nutrition session involved practical exercises using real food and scales to estimate and measure portions, fostering active learning. In the laboratory sessions, students engaged in hands‑on activities such as the extraction of essential oils and the preparation of soaps, candles, and herbal salves—offering a rare opportunity to apply theoretical knowledge in a practical setting. These components promoted deep engagement and immersion in integrative health practices.

In the online version, although hands‑on activities were not feasible, the course maintained interactivity through weekly quizzes, live sessions every Friday, and short assignments aimed at encouraging students to reflect on the practical relevance of each topic. Students had access to recorded lectures and support materials hosted on a Google Site platform, integrated with Slido, Google Drive, and other tools. This solution proved to be effective and demonstrates that it is possible to deliver engaging, participatory online training with limited resources [11]. Future editions, however, could benefit from hosting the course on more structured platforms (e.g., Moodle) and incorporating elements such as peer assessment to further improve learning outcomes.

Despite its success, the course revealed an important shortcoming: the lack of mechanisms to accommodate students with disabilities, both in‑person and online. This limitation is reflective of a broader issue in the education system, where short‑term courses often overlook the need for accessibility. Future editions should prioritize inclusive practices, accessible content formats, and assistive technologies.

The course also sparked important discussions regarding the need to raise awareness of the existence and value of integrative medicine. Many health professionals remain unfamiliar with this field, despite its growing relevance. In practice, patients in Mozambique frequently use complementary and alternative therapies [12–17], yet medical professionals are often untrained to address such disclosures. There is a lack of knowledge on the safety, efficacy, and proper integration of complementary therapies into clinical care. Furthermore, the prevailing biomedical model often overlooks holistic approaches, which are essential to person‑centered healthcare.

In Mozambique, research on this subject remains scarce. As such, this course not only fills a critical educational gap but also constitutes the first documented training initiative in the field of integrative medicine in the country. It represents an initial, yet meaningful, step towards advancing both academic and practical understanding of these practices within the national health system.

The positive reception of the course by the faculty, department, and university further reinforces its relevance and potential for institutionalization. The laboratory equipment and materials acquired for the course will enable the Faculty of Sciences to continue offering similar training in the future, thereby ensuring sustainability and integration of these contents into undergraduate and postgraduate programs. This contributes to the long‑term objective of embedding integrative medicine into the formal medical education system, both in Mozambique and potentially across other African countries.

## Conclusion

This pioneering course in integrative medicine has demonstrated both feasibility and effectiveness in the Mozambican context. It successfully reached diverse audiences—including undergraduate and postgraduate students, underscoring the growing demand for structured education in this area. The overall high satisfaction rates, meaningful learning outcomes, and strong completion rate attest to the relevance and quality of the course design.

Both the in‑person and online modalities proved effective. The face‑to‑face sessions allowed for deeper experiential learning, particularly through practical activities such as laboratory work and clinical simulations. The online version, although developed and implemented in a short timeframe and without access to a formal learning management system, still managed to maintain student engagement and foster reflective learning through interactive tools and live sessions. This confirms that impactful educational experiences in integrative medicine can be delivered through multiple formats, even in low‑resource settings.

Importantly, this initiative marks a foundational step for the institutionalization of integrative medicine in Mozambique. Beyond providing knowledge and practical skills, the course contributed to strengthening critical thinking among future and current health professionals about the role of traditional, complementary, and integrative practices in healthcare. As the first documented initiative of its kind in the country, it establishes a model for replication and scaling, not only within Mozambique but also across other African settings where similar educational gaps persist. Sustained investment in similar initiatives, coupled with the integration of these topics into medical curricula, will be crucial for advancing more holistic, culturally attuned, and patient‑centered approaches to healthcare in the region.

## Additional Files

The additional files for this article can be found as follows:

Supplementary File 1. Detailed program for both the first and second editions of the course are provided in.

Supplementary File 2. Expanded sociodemographic data, academic background, CAM training, and learning preferences.

This finding of multiple local introgression events has at least three key implications.

## References

[r1] Somerville C, Munguambe K. The rise of non‑communicable disease (NCDs) in Mozambique: Decolonising gender and global health. Gender Dev. 2021;29(1):189–206. doi:10.1080/13552074.2021.1885220.

[r2] Institute for Health Metrics and Evaluation (IHME). Global Burden of Disease Study 2019 (GBD 2019) results. Published October, 2020. https://vizhub.healthdata.org/gbd-results/.

[r3] Takeyama N, Muzembo BA, Jahan Y, Moriyama M. Health‑seeking behaviors in Mozambique: A mini‑study of ethnonursing. Int J Environ Res Public Health. 2022;19(4):2462. doi:10.3390/ijerph19042462. 35206649 PMC8872320

[r4] Barbosa F, Hlashwayo D, Sevastyanov V, et al. Medicinal plants sold for treatment of bacterial and parasitic diseases in humans in Maputo city markets, Mozambique. BMC Complement Med Ther. 2020;20(1):19. doi:10.1186/s12906-019-2809-9. 32020866 PMC7076832

[r5] Aparicio H, Hedberg I, Bandeira S, Ghorbani A. Ethnobotanical study of medicinal and edible plants used in Nhamacoa area, Manica province–Mozambique. S Afr J Bot. 2021;139:318–328. doi:10.1016/j.sajb.2021.02.029.

[r6] Moura I, Duvane JA, Silva MJ, Ribeiro N, Ribeiro‑Barros AI. Woody species from the Mozambican Miombo woodlands: A review on their ethnomedicinal uses and pharmacological potential. J Med Plants Res. 2018;12(2):15–31. doi:10.5897/JMPR2017.6540.

[r7] Bruschi P, Morganti M, Mancini M, Signorini MA. Traditional healers and laypeople: A qualitative and quantitative approach to local knowledge on medicinal plants in Muda (Mozambique). J Ethnopharmacol. 2011;138(2):543–563. doi:10.1016/j.jep.2011.09.055. 22008876

[r8] Bruschi P, Mancini M, Mattioli E, Morganti M, Signorini MA. Traditional uses of plants in a rural community of Mozambique and possible links with Miombo degradation and harvesting sustainability. J Ethnobiol Ethnomed. 2014;10:59. doi:10.1186/1746-4269-10-59. 25056487 PMC4112836

[r9] Bandeira SO, Gaspar F, Pagula FP. African ethnobotany and healthcare: Emphasis on Mozambique. Pharm Biol. 2001;39(Suppl 1):70–73. doi:10.1076/phbi.39.s1.70.0002. 21554173

[r10] Ring M, Mahadevan R. Introduction to integrative medicine in the primary care setting. Prim Care. 2017;44(2):203–215. doi:10.1016/j.pop.2017.02.006. 28501225

[r11] Ben‑Arye E, Keshet Y, Schiff A, et al. From COVID‑19 adversity comes opportunity: Teaching an online integrative medicine course. BMJ Support Palliat Care. 2024;14:e1547–e1555. doi:10.1136/bmjspcare-2020-002713. 34266910

[r12] Machava NE, Mulaudzi FM, Salvador EM. Household factors of foodborne diarrhea in children under five in two districts of Maputo, Mozambique. Int J Environ Res Public Health. 2022;19(23):15600. doi:10.3390/ijerph192315600. 36497675 PMC9739694

[r13] Sitoe E, Van Wyk BE. An inventory and analysis of the medicinal plants of Mozambique. J Ethnopharmacology. 2024;319(Pt 2):117137. doi:10.1016/j.jep.2023.117137. 37783405

[r14] Camille L, Bamba G, Ibrahima Bara D, et al. Use of traditional medicine and control of hypertension in 12 African countries. BMJ Global Health. 2022;7(6):e008138. doi:10.1136/bmjgh-2021-008138. PMC9163537 35654446

[r15] Farooq H, Bero C, Guilengue Y, et al. Snakebite incidence in rural sub‑Saharan Africa might be severely underestimated. Toxicon. 2022;219:106932. doi:10.1016/j.toxicon.2022.106932. 36181779

[r16] Gove JIM, Giordani RCF, Jasper VHdS, Estavela A, Bezerra I. “Xilala is only treated with a good hand”: A study on the treatment of malnutrition in Mozambique. Cad Saúde Pública. 2021;37:e00212320. doi:10.1590/0102-311X00212320. 34406215

[r17] Groh K, Audet CM, Baptista A, et al. Barriers to antiretroviral therapy adherence in rural Mozambique. BMC Public Health. 2011;11:650. doi:10.1186/1471-2458-11-650. 21846344 PMC3171727

