# Supplementary material for: Transforming Healthcare: Mozambique’s Pioneering Integrative Medicine Course
Source: Ann Glob Health. 2026 Feb 12;92(1):15. doi: 10.5334/aogh.4785 (PMC12904132; doi:10.5334/aogh.4785)
Supplement: Supplementary File 1. — Detailed program for both the first and second editions of the course are provided in. [file agh-92-1-4785-s1.pdf]

Supplementary File 1. Detailed program for both the first and second editions of the course are provided in

1ST EDITION: SEPTEMBER 16-20, 2024

Time: 8:00 AM - 1:00 PM
Location: Room A7 - Department of Biological Sciences - Faculty of Sciences, UEM, Main Campus (Av. Julius Nyerere no. 3453, Maputo)

Time	Topic
**DAY 1 [Monday] - 16/09/2024**	
08:00-09:00	Introduction and Pre-test<br>Introduction to Integrative Medicine: Definition and Context<br>History of Integrative Medicine: Transition from an Alternative Model to an Integrative Medicine Model
09:05-10:00	Types of Complementary and Alternative Therapies: A. Mind-body therapies, B. Biological practices, C. Manipulative and body-based practices, D. Energy healing therapies, E. Integrative medical systems
10:00-10:15	*Snack Break*
10:15-11:00	Evaluation of the state of research evidence in integrative medicine and reliable sources<br>Planning an integrative treatment plan: challenges, role of health professionals, ethics, and legislation
11:05-12:00	The role of guided imagery in health and healthcare, and how it can be used both professionally and personally<br>What is Mindfulness – and what it is not? Ways to practice it. Why meditate? Benefits of mindfulness
12:05-13:00	Relaxation techniques
**DAY 2 [Tuesday] - 17/09/2024**	
08:00-09:00	Introduction to African and Mozambican Traditional Medicine: Historical and cultural context, key principles, and therapeutic approaches
09:05-10:00	Traditional practices and common therapies in Africa, their application in health and precautions
10:00-10:15	*Snack Break*
10:15-11:00	The role of anthropology and ethnobotany in the study of African and Mozambican Traditional Medicine
11:05-12:00	Case study discussion on the use of traditional medicine and integration with other therapeutic practices<br>Challenges of validating African traditional medicine and its integration into the national health system
12:05-13:00	Role-play exercise: How to discuss the use of African traditional medicine with a patient
**DAY 3 [Wednesday] - 18/09/2024**	
08:00-09:00	Introduction to Phytotherapy: Definition and basic principles, history, and evolution of medicinal plant use<br>Identification of Medicinal Plants; Monographs of medicinal plants, medicinal properties, precautions, and contraindications
09:05-10:00	Evaluation of the efficacy and quality of phytopharmaceuticals<br>Clinical applications and use of phytopharmaceuticals; integration with other therapies<br>European, Brazilian, and Mozambican phytotherapy legislation
10:00-10:15	*Snack Break*
10:15-11:00	Self-medication and its challenges: the use of herbal remedies in health emergencies among health students in Mozambique; potential for drug interactions and precautions

Time	Topic
11:05-12:00	Laboratory Class - Extraction and phytochemical analysis of plant extracts; Preparation of ointments based on plants
12:05-13:00	Laboratory Class - Extraction and phytochemical analysis of plant extracts (continued)
**DAY 4 [Thursday] - 19/09/2024**	
08:00-09:00	Introduction to Aromatherapy: Definition and history<br>How essential oils are obtained, their composition and properties<br>Main essential oils used in aromatherapy and essential oil monographs<br>Evidence base for aromatherapy and precautions
09:05-10:00	Laboratory Class - Extraction of essential oils. Production of candles and soaps with essential oils
10:00-10:15	*Snack Break*
10:15-11:00	Laboratory Class - Extraction of essential oils. Production of candles and soaps with essential oils (continued)
11:05-12:00	Laboratory Class - Extraction of essential oils (continued)
12:05-13:00	Introduction to Nutrition and Emotional Well-being
**DAY 5 [Friday] - 20/09/2024**	
08:00-09:00	Nutrition and Emotional Well-being (continued)
09:05-10:00	Nutrition and Emotional Well-being (continued)
10:00-10:15	*Snack Break*
10:15-11:00	Nutrition and Emotional Well-being (continued)
11:05-12:00	Final Considerations on the Integrative Medicine Course and Focus Group Discussion
12:05-13:00	Final Exam, Course Evaluation, Post-test, and Certificate Distribution

2ND EDITION (ONLINE)

COORDINATION:
University Eduardo Mondlane – Faculty of Sciences – Department of Biological Sciences

OBJECTIVES:
To provide participants with a comprehensive and practical understanding of integrative approaches to health and well-being promotion.

TARGET AUDIENCE:
Medical students and those in related health fields.

PROGRAM [2025]:

Week 1 [January 6-10] – Introduction to Integrative Medicine and Complementary Therapies
Week 2 [January 13-17] – Scientific Evidence in Integrative Medicine and Mental Well-being
Week 3 [January 20-24] – African and Mozambican Traditional Medicine
Week 4 [January 27-31] – Phytotherapy and Medicinal Plant Use
Week 5 [February 3-7] – Aromatherapy
Week 6 [February 10-14] – Laboratory Demonstrations of Phytotherapy and Aromatherapy
Week 7 [February 17-21] – Nutrition and Emotional Well-being
February 27 (Thursday) – Final Evaluation

The classes will be available in recorded format, with an average duration of 1.5 hours per week. Additionally, the content will be complemented by discussion forums and other activities, such as readings, practical exercises, quizzes, and more.

LIVE WEEKLY SESSIONS:
Fridays from 7:00 PM to 7:40 PM on Google meet.

KNOWLEDGE ASSESSMENT AND CERTIFICATES:

- At the end of the course, there will be a Knowledge Assessment through a multiple-choice exam.
- The Knowledge Assessment will take place on a single date and will only be valid for students who participated in the activities guided by facilitators each week (including the Discussion Forum). Approval will be granted to participants who achieve 50% or more correct answers.
- A Certificate of Attendance will be awarded to participants who meet the above criteria.
