# Supplementary material for: Transforming Healthcare: Mozambique’s Pioneering Integrative Medicine Course
Source: Ann Glob Health. 2026 Feb 12;92(1):15. doi: 10.5334/aogh.4785 (PMC12904132; doi:10.5334/aogh.4785)
Supplement: Supplementary File 2. — Expanded sociodemographic data, academic background, CAM training, and learning preferences. [file agh-92-1-4785-s2.pdf]

Supplementary File 2. Expanded Sociodemographic data, academic background, CAM Training, and learning preferences

Table 1. Participant characteristics and previous exposure to Complementary and Alternative Therapies

	1st Edition (n=29)	2nd Edition (n=105)	Total (n=134)
**Age range**			
18–24	23 (79.3%)	81 (77.1%)	104 (77.6%)
25–34	6 (20.7%)	22 (21.0%)	28 (20.9%)
35–44	0 (0.0%)	2 (1.9%)	2 (1.5%)
**Current degree program**			
Medicine	24 (82.8%)	58 (55.2%)	82 (61.2%)
Dentistry	0 (0.0%)	2 (1.9%)	2 (1.5%)
Bachelor's in Speech Therapy	1 (3.5%)	0 (0.0%)	1 (0.8%)
Bachelor's in Physiotherapy	0 (0.0%)	1 (1%)	1 (0.8%)
Bachelor's in Public Health	0 (0.0%)	5 (4.8%)	5 (3.7%)
Bachelor's in Nutrition	1 (3.5%)	3 (2.9%)	4 (3.0%)
Bachelor's in Biology and Health	2 (6.9%)	20 (19.1%)	22 (16.4%)
Bachelor's in Clinical and Laboratory Analysis	0 (0.0%)	1 (1%)	1 (0.8%)
Bachelor's in Biomedical Laboratory Technology	0 (0.0%)	5 (4.8%)	5 (3.7%)
Bachelor's in Psychology	1 (3.5%)	4 (3.8%)	5 (3.7%)
Bachelor's in Pharmacy	0 (0.0%)	1 (1%)	1 (0.8%)
Bachelor's in Nursing	0 (0.0%)	1 (1%)	1 (0.8%)
Technical Nursing in Maternal and Child Health	0 (0.0%)	1 (1%)	1 (0.8%)
Master's in Nutritional Science	0 (0.0%)	1 (1%)	1 (0.8%)
Master's in Maternal, Neonatal, and Child Health	0 (0.0%)	1 (1%)	1 (0.8%)
PhD in Biosciences	0 (0.0%)	1 (1%)	1 (0.8%)
**Institution**			
Eduardo Mondlane University (UEM)	21 (72.4%)	43 (41.0%)	64 (47.8%)
ISCTEM	5 (17.2%)	40 (38.1%)	45 (33.6%)
ISCISA	2 (6.9%)	10 (9.5%)	12 (9%)
Pedagogical University of Maputo	1 (3.5%)	1 (1%)	2 (1.5%)
UniLúrio	0 (0.0%)	2 (1.9%)	2 (1.5%)
University of Algarve	0 (0.0%)	1 (1%)	1 (0.8%)
Instituto Superior Monitor	0 (0.0%)	1 (1%)	1 (0.8%)
USTM	0 (0.0%)	5 (4.8%)	5 (3.7%)
IFOPTEC – Institute of Polytechnic Training and Technologies	0 (0.0%)	1 (1%)	1 (0.8%)
Institute of Management and Entrepreneurship Guaza Muthini	0 (0.0%)	1 (1%)	1 (0.8%)
**Have you received training in any of the following complementary/alternative therapies?**			
Dietary supplements	1 (3.4%)	18 (17.1%)	19 (14.2%)
Meditation	1 (3.4%)	9 (8.6%)	10 (7.5%)
Herbal medicine	0 (0.0%)	6 (5.7%)	6 (4.5%)
Aromatherapy	0 (0.0%)	3 (2.9%)	3 (2.2%)
Acupuncture	0 (0.0%)	2 (1.9%)	2 (1.5%)
Cognitive-behavioral therapy	0 (0.0%)	1 (1.0%)	1 (0.7%)
Music therapy	0 (0.0%)	1 (1.0%)	1 (0.7%)
Speech therapy	1 (3.4%)	0 (0.0%)	1 (0.7%)
**Which complementary/alternative therapies have you used?**			
Dietary supplements	5 (17.2%)	11 (10.5%)	16 (11.9%)
Meditation	4 (13.8%)	10 (9.5%)	14 (10.4%)
Herbal medicine	2 (6.9%)	10 (9.5%)	12 (9%)
Acupuncture	0 (0.0%)	1 (1%)	1 (0.7%)
Aromatherapy	0 (0.0%)	1 (1%)	1 (0.7%)
Cognitive-behavioral therapy	0 (0.0%)	1 (1%)	1 (0.7%)
Speech therapy	1 (3.4%)	0 (0.0%)	1 (0.7%)

Table 2. Topics and Contents Considered Most Relevant by Participants

Topic	1st Edition (n=85 mentions)	2nd Edition (n=116 mentions)	Total Mentions (n=201)
Phytotherapy	24 (28.2%)	19 (16.4%)	43 (21.4%)
Traditional medicine	1 (1.2%)	27 (23.3%)	28 (13.9%)
Mental well-being	15 (17.7%)	12 (10.3%)	27 (13.4%)
Nutrition	17 (20.0%)	10 (8.6%)	27 (13.4%)
Aromatherapy	22 (25.9%)	4 (3.5%)	26 (12.9%)
Various complementary and alternative therapies	6 (7.1%)	11 (9.5%)	17 (8.5%)
Scientific evidence	0 (0.0%)	16 (13.8%)	16 (8.0%)
Introductory concepts of integrative medicine	0 (0.0%)	11 (9.5%)	11 (5.5%)
Laboratory classes on phytotherapy and aromatherapy	0 (0.0%)	6 (5.2%)	6 (3.0%)

***Note**: The denominator in this table refers to the number of mentions (n=201), not the number of students, since some students listed more than one relevant topic*.

Table 3. Preferred teaching format – condensed overview

Format	1st Edition (n=65)	2nd Edition (n=162)	Overall (n=227)
Active and participatory learning	55 (84.6%)	88 (54.3%)	143 (63.0%)
Lectures	10 (15.4%)	67 (41.4%)	77 (33.9%)
Resources	0	7 (4.3%)	7 (3.1%)

Table 4. Preferred teaching format – expanded breakdown

Activity or Modality	1st Edition (n=65)	2nd Edition (n=162)	Total Mentions (n=227)
Lectures	10 (15.4%)	67 (41.4%)	77 (33.9%)
Practical activities or demonstrations	30 (46.2%)	37 (22.8%)	67 (29.5%)
Live sessions	0	27 (16.7%)	27 (11.9%)
Group discussions or debates	21 (32.3%)	0	21 (9.3%)
Assignments	0	12 (7.4%)	12 (5.3%)
Resources	0	7 (4.3%)	7 (3.1%)
Quizzes	0	7 (4.3%)	7 (3.1%)
Web interaction	0	5 (3.1%)	5 (2.2%)
Case studies	4 (6.2%)	0	4 (1.8%)

***Note**: Students could select more than one preferred format or activity, so the total reflects the number of mentions, not unique individuals.*
